# A rapid *in vitro* methodology for simultaneous target discovery and antibody generation against functional cell subpopulations

**DOI:** 10.1038/s41598-018-37462-1

**Published:** 2019-01-29

**Authors:** Allison M. L. Nixon, Alejandro Duque, Nicholas Yelle, Megan McLaughlin, Sadegh Davoudi, Nicolas M. Pedley, Jennifer Haynes, Kevin R. Brown, James Pan, Traver Hart, Penney M. Gilbert, Sheila K. Singh, Catherine A. O’Brien, Sachdev S. Sidhu, Jason Moffat

**Affiliations:** 1 Donnelly Centre, University of Toronto, Toronto, M5S 3E1 Canada; 2 Department of Molecular Genetics, University of Toronto, Toronto, M5S 1A8 Canada; 3 Stem Cell and Cancer Research Institute, McMaster University, Hamilton, L8S 4K1 Canada; 4 Department of Biochemistry and Biomedical Sciences, McMaster University, Hamilton, L8N 3Z5 Canada; 5 Institute of Biomaterials and Biomedical Engineering, University of Toronto, Toronto, M5S 3G9 Canada; 6 Princess Margaret Cancer Centre, University Health Network, Toronto, M5G 2MC1 Canada; 7 Department of Surgery, Faculty of Health Sciences, McMaster University, Hamilton, L8N 3Z5 Canada; 8 Department of Surgery, Toronto General Hospital, Toronto, M5G 2C4 Canada; 9 Department of Laboratory Medicine and Pathobiology, University of Toronto, Toronto, M5S 1A1 Canada; 10 Canadian Institute for Advanced Research, Toronto, M5G 1Z8 Canada; 11 Present Address: Department of Bioinformatics and Computational Biology, MD Anderson Cancer Center, Houston, Texas 77030 USA

## Abstract

Cell surface antigen discovery is of great interest for biomedical research both for isolation of rare cell populations and therapeutic targeting. We developed a rapid, cost-effective, fully *in vitro* technology which facilities the simultaneous target discovery and human antibody generation on the surface of virtually any cell population of interest. We apply our technique to human colorectal cancer-initiating cells (CICs) and identify hundreds of unique human antibodies. We characterized the top three antibody candidates targeting these CICs and identify their protein targets as integrin α7 (ITGA7), HLA-A1 and integrin β6 (ITGB6). We demonstrate that these antibodies can be used to isolate self-renewing colorectal CICs, and that the integrin α7 antibody can prospectively identify glioblastoma brain tumor initiating cells as well as human muscle stem cells. We also demonstrate that genetic ablation of integrin β6 impedes colorectal CIC function. The methodology can be readily applied to other cell populations including stem cells, cancer, or immune cells to facilitate the rapid identification of novel targets and simultaneous generation of potent and specific antibodies with therapeutic potential.

## Introduction

Cell surface target discovery is of great interest for biomedical research. Surface protein targets can be exploited to kill, isolate, or augment the function of virtually any cell population of interest using affinity reagents including monoclonal antibodies, antibody drug conjugates (ADCs), peptides and bi-specific antibodies for engaging immune cells such as T-cell engagers (BiTEs). The application of these technologies in the clinic is limited by lack of efficacious epitopes on clinically-relevant cell populations. Most methods of cell population-specific target discovery rely on transcriptomics, proteomics or functional genetics. Each of these strategies may yield a list of genes/proteins likely to be important for a specific cell population, however, none of these strategies results in the generation of a research tool and potentially translatable reagent, such as an antibody. We propose that coupling target discovery to antibody generation can speed up the process from diseased cell population of interest, to research tool and targeting agent.

Animal adaptive immune systems have been repeatedly exploited for the purpose of antibody generation and also target discovery^1^. In one classic example, seeking novel hematopoietic stem cell makers, researchers immunized a naïve mouse with CD34+ hematopoietic stem cells^2^. The animal mounted an adaptive immune response, and its splenocytes were subsequently isolated and immortalized by fusion to multiple myeloma cells. Supernatants from the resulting hybridomas were screened, and AC133 was identified as specific for the cell population of interest^2^. The target of AC133 was later identified as the penta-span transmembrane glycoprotein, CD133^3^, which has become one of the most prolific stem and cancer-initiating cell (CIC) markers^4–8^. More recently, the AC133 antibody was partially humanized by fusing the mouse variable domains from the original hybridoma with human constant domains to create a chimeric antibody. Chimeric AC133, as well as other humanized monoclonal antibodies against CICs, have shown significant anti-tumor effects in preclinical models, providing evidence that such CIC markers may also be good therapeutic targets^9^. Although animal-reliant strategies for antibody discovery and development have been highly successful, they are time consuming, resource intensive, and requires a great deal of expertise and labor, taking up to half a year until an antibody is purified^1^ and much longer to develop humanized versions suitable for clinical applications.

Advancements in synthetic biology and protein engineering have led to the development of *in vitro* yeast- and phage-displayed synthetic antibody libraries that exceed the naïve diversities of natural immune repertoires^10,11^. The physical linkage between the genotype (i.e. the sequence of antibody variable regions) and phenotype (i.e. binding specificity) in display systems serves as a barcoding system that can be leveraged together with deep sequencing for cost-effective broad screening capabilities^12–14^. Synthetic libraries have permitted the rapid and effective development of many highly specific, fully human antibodies against purified recombinant antigens and antigens expressed in their native forms on the cell surface^12–14^. Individual antibody binders can be cloned or synthesized from these pools in less than a week, and in parallel, pools of binders specific for a population of interest can be deep sequenced. Recently, an alternative method has been described that uses transient transfection of alternating host cell lines and stringent washing steps for biopanning with naïve phage-displayed single-chain variable fragment libraries^15^. Herein, we describe a novel approach termed CellectAb, inspired by the animal immunization technique for marker discovery, that links target discovery to *in vitro* synthetic antibody generation.

Most hematological and solid malignancies have been found to comprise functionally diverse subpopulations of cells that differ in their potential for proliferation, self-renewal, therapy resistance and metastasis formation^16–21^. This heterogeneity presents major challenges to both diagnosis and treatment, positioning itself as one of the next frontiers in cancer biology^22,23^. One aggressive subpopulation of cells can be functionally referred to as cancer-initiating cells (CICs), based on their functional ability to initiate a tumour spheroid *in vitro*, or a tumour *in vivo*. Despite initial success using markers like CD133 to isolate, study and target CICs across many cancer types, including colorectal carcinoma (CRC), conflicting reports suggest that CD133 and other current markers are insufficient to reliably purify CICs^4,8^. This also may be indicative that the underlying biology of these CD133^high^ subpopulations is incompletely understood. It has been repeatedly shown, particularly in the blood system, that cell populations homogeneous for certain markers, but remaining functionally heterogeneous, can be further dissected with the discovery of new markers that fractionate into biologically distinct subpopulations^24^. Thus, novel antigens on the surface of CD133^high^ CRC CICs may serve as markers of aggressive cell populations, and as therapeutic targets.

To this end, we designed and implemented a novel selection strategy to isolate human synthetic antibody fragments specific to a subpopulation of CD133^high^ human CRC CICs, and identified hundreds of CIC-specific binders by deep sequencing. We characterized three candidates as binding specifically to the CRC CIC population, and identified their targets as integrin α7 (ITGA7), HLA-A1 and integrin β6 (ITGB6). Furthermore, we demonstrate that these antibodies can be used to enrich for self-renewing colorectal CICs, and that the integrin α7 antibody can be used to identify a subpopulation of cells from glioblastoma capable of tumor initiation and human muscle stem cells. We further observed that genetic ablation of integrin β6 impedes CRC CIC function, linking ITGB6 to CIC biology for the first time. Our CellectAb methodology can be readily applied to other cell subpopulations to facilitate the rapid identification of novel targets and simultaneous generation of potent and specific antibodies.

## Results

### Cell panning for presumptive colorectal CIC markers using AC133 cell populations

**Figure 1:**
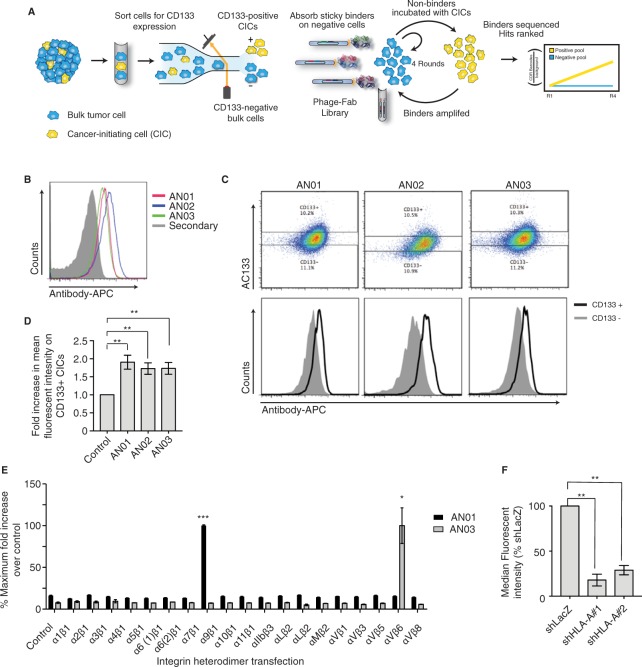
CellectAb antibody screening methodology and antigen identification. (**A**) Schematic of phage-displayed synthetic antibody selections using FACS sorted AC133^high^ CICs as positive selection, and AC133^low^ bulk cells as negative selection (see Methods for details). (**B**) Histograms from a representative flow cytometry experiment showing binding of AN01, AN02 and AN03 to POP92 cells. (**C**) Flow cytometry analysis from a representative experiment of POP92 co-stained with CD133-FITC and AN0#-APC (top). Histograms comparing the AN0# binding on the CD133^high^ to CD133^low^ populations gated above (bottom). (**D**) Bar chart illustration of the mean fold increase in AN01, AN02 or AN03 mean fluorescent intensity (MFI) on CD133^high^ cells compared to CD133^low^ (normalized to 1 in each experiment). Error bars are standard error of the mean (SEM). For AN01 N = 7, for AN02 N = 6, and for AN03 N = 7 biological replicates. **(E**) Bar chart illustration of percent maximum AN01 and AN03 binding on HEK293T cells co-transfected with various integrin subunit pairs relative to in-tube control (see Methods for details). Error bars are SEM from N = 3 separate transfections. Significance was calculated for each transfection compared to control transfection by unpaired student’s t-test. (**F**) Bar chart illustrating AN02 binding to POP92 cells stably transduced with shRNA against *HLA-A* or shLacZ negative control. Data is represented as % shLacZ MFI, error bars are SEM, N = 3 independent transductions. Significance was calculated using two-tailed paired t-test. *p < 0.05, **p < 0.01, ***p < 0.001.

We sought to couple unbiased target and antibody discovery to a functional subpopulation of cancer cells aiming to identify antibodies for marking and potentially therapeutically targeting these cells. We exploited a patient-derived colorectal cancer (CRC) cell spheroid model known as POP92, which maintains cellular heterogeneity and stem-like properties, and from which the AC133 epitope can be used to enrich for functional CICs^25^. To isolate the CIC population, spheroids were dissociated to a single cell suspension, stained with AC133 and sorted by FACS into CD133^high^ presumptive CICs and a CD133^low^ bulk cell population (Fig. 1A). In order to generate antibodies against native antigens, we used live cells for our selection procedure. Over an eight-hour period and under low flow pressure, we were able to isolate by FACS at least 2 × 10^6^ CD133^high^ CICs and 4 × 10^6^ CD133^low^ bulk cells, representing the top 10% and bottom 20% of the entire cell population, respectively. The CD133^low^ cells were split in half and used for the negative enrichment, and to deplete non CIC-specific binders in the positive enrichment, as detailed below.

There are a number of existing published and proprietary human synthetic antibody libraries. These libraries have shown great utility for the generation of highly specific and potent antibodies against a wide variety of antigens. However, most of these libraries are not optimized for deep sequencing strategies. Observations from the repeated application of these libraries suggest that the contributions of only two complementarity determining regions CDRs (H3 and L3) largely dictates binding specificity and affinity^26,27^. Based on this, and to facilitate ease of deep sequencing and downstream cloning applications, we designed a custom high-complexity library of fully human antigen binding fragments (Fabs) displayed on M13 bacteriophage wherein sequence diversity is restricted to two CDRs H3 and L3 (Fig. S1). To obtain high-confidence binders specific for the CD133^high^ presumptive CIC population from this library, we performed sequential rounds of selection enriching for binders to the CIC population. In brief, we incubated the naïve library with CD133^low^ cells to deplete non-specific binders from the library, and transferred non-binding phage Fab to CD133^high^ presumptive CICs. CIC-binding phage-Fabs were subsequently amplified in an *E. coli* host, and subjected to three additional rounds of the same selection process (positive enrichment Fig. 1A). In parallel, we selected for binders on the bulk CD133^low^ cells to serve as a control (negative enrichment Fig. 1A). Through these repeated rounds of selections, Fab-bearing phages with binding specificity for the CD133^high^ population became enriched in the positive enrichment pool and depleted in the negative pool. The sequence of each of the CDRs for each Fab acts as a molecular barcode, which tracks the protein sequence, binding specificity and unique characteristics of each Fab. After the final round of selection, genomic DNA from each of the Fab-phage selection pools was isolated and deep sequencing was performed on the CDRs. We calculated a sequence-based enrichment score, based on the proportion of sequence reads in the positive compared to negative pool, and identified seven unique Fab sequences for characterization studies (Table 1). The most prevalent seven Fabs were named as AN01–AN07, based on order of enrichment.

**Table 1:** Identification of enriched Fab sequences.

Antibody ID	Read count (+pool)	Enrichment score
AN01	579593	18.61
AN02	15347	13.37
AN03	12736	13.1
AN04	10027	12.75
AN05	5187	11.8
AN06	1579	10.09
AN07	1182	9.67

Phage-Fab output pools from the positive enrichment and negative enrichment were collected after the fourth round of selection. Complementarity determining regions (CDRs) were PCR amplified and deep sequenced. Total number of reads in the positive (+) enrichment is shown in column 2, and column 3 shows the enrichment score calculated as: log2 positive read counts/log2 negative (−) enrichment read counts. See Table S1 for full list.

### Antibodies bind preferentially to the CD133^high^ POP92 population

We tested the binding of these seven Fabs to POP92 cells by flow cytometry and found that AN01, AN02 and AN03 bound robustly to the cell surface (Fig. 1B). To test if these three antibodies recognize specifically the CD133^high^ CICs, we co-stained pairwise with each Fab and AC133 and analyzed the top and bottom 10% of CD133-expressing cells for Fab mean fluorescence intensity (MFI) (Fig. 1C). The observed MFIs for AN01, AN02 and AN03 were significantly higher on the CD133-positive population compared to the CD133-negative population (Fig. 1D p < 0.01 Unpaired t-test with Welch’s correction, N = 7), validating the ability of the selection strategy to yield antibodies with preferential specificity for the CD133^high^ CIC subpopulation.

### AN01, AN02 and AN03 recognize distinct cell surface antigens on a variety of cell types

To understand if the binders AN01, AN02 or AN03 recognize distinct surface targets, we first test their binding to CD133. We mixed HEK293 cells engineered to express CD133-GFP fusion protein and matched empty-vector control in a 1:1 ratio and stained with each Fab. The matched lines were gated separately by flow cytometry based on GFP expression, and then assessed for Fab binding. We observed no change in Fab MFI between the CD133 overexpression line (GFP+) and negative control (GFP−), indicating that the Fabs do not bind to the CD133 protein directly (Fig. S2A,B). We next characterized by flow cytometry the binding patterns of each Fab on a panel of cell lines from different cancer types, as well as noncancerous cell types. We observed distinct binding patterns for each Fab that did not correlate with CD133 expression status (Table S1). Taken together, this indicated that AN01, AN02 and AN03 do not bind directly to CD133 and each binds to a distinct cell surface antigen. Each Fab was reformatted and purified as a bivalent single-chain IgG, and similar binding patterns were observed (data not shown).

### Identification of the antigens recognized by AN01, AN02 and AN03

We next aimed to identify the cell-surface antigen for each antibody by immunoprecipitation (IP) followed by mass spectrometry (IP-MS). POP92 cell lysates were incubated with either AN01, AN02, or AN03 antibodies and interacting proteins were isolated by IP and identified by mass spectrometry. AN01 specifically immuno-precipitated integrin subunits alpha(α)7 and beta(β)1 (Table 2). Together, these subunits are known to form the heterodimeric laminin receptor and cell adhesion molecule, integrin α7β1. AN02 uniquely pulled down human leukocyte antigen (HLA) major histocompatibility complex (MHC) class I antigen A-1 (HLA-A1 encoded at the *HLA-A* locus)(Table 2). MHC Class I molecules are cell surface receptors expressed on all nucleated cells that act as part of the adaptive immune system to present cytoplasmic peptides to immune surveillance cells. In the case of infection or cancer, these peptides may be identified by cytotoxic T-cells as “non-self”, targeting the aberrant cell for destruction. There are over two thousand unique HLA-A haplotypes broken down by serotype (i.e. HLA-A1, HLA-A2), and further by allele. Each diploid cell expresses up to two unique haplotypes from the *HLA-A* locus, and these are used for tissue matching for transplantation^28^. The majority of HLA-A peptides identified represent the HLA-A1 serotype, suggesting that AN02 is a serotype specific binder for HLA-A1, not other HLA-A serotypes. Lastly, AN03 specifcally immunoprecipitated integrin subunits αV and β6, representing the integrin αVβ6 heterodimer (Table 2). This heterodimeric cell adhesion molecule acts as a receptor for fibronectin, vitronectin and Latency-Associated Peptide bound TGFβ (LAP-TGFβ)^29,30^. Thus, the putative targets for AN01, AN02 and AN03 were integrin α7β1, HLA-A1, and integrin αVβ6, respectively.

**Table 2:** Identification of targets of *AN02* and *AN03* by *IP-MS*.

Protein	Description	BOC	AN01	AN02	AN03
ITGA7	Integrin α7	0	31	0	0
ITGB1	Integrin β1	0	25	0	1
HLA-A1	HLA-A, serotype1	0	0	33	2
ITGAV	Integrin αV	0	0	0	2
ITGB6	Integrin β6	0	0	0	2

POP92 whole cell lysate was incubated with scIgG, followed by Protein G bead pull down. BOC is beads only control. Peptide counts for top hits are shown. See Table S2 for full results.

### Validation of the protein targets of AN01, AN02 and AN03

To further confirm the targets of these three antibodies, we tested if various perturbations to each prospective gene target could affect binding. First, we used RNA interference (RNAi) to knock down the gene expression in POP92 cells and assessed antibody binding by flow cytometry. We observed dramatic reductions in binding of AN01, AN02 and AN03 to POP92 cells stably transduced with two independent short hairpins RNAs (shRNAs) against each of their respective targets, compared to control non-targeting shRNA (Figs 1F and S2F–I). A panel of integrin knockout HAP1 cell lines also confirmed AN01 for integrin α7β1 specificity (Fig. S2J).

The integrin family is composed of 18 α and 8 β subunits that combine specifically to form 24 distinct αβ heterodimers^31^ (Fig. S2E). The human integrins show a high degree of sequence homology between members of the α and β subunit families, likely due to gene expansion from a common evolutionary ancestor^32^. To understand the specificity of AN01 and AN03 for integrin heterodimer recognition, we overexpressed twenty different integrin heterodimers by transfection in HEK293T cells and assessed AN01 and AN03 binding by flow cytometry. Remarkably, the results revealed a very high degree of specificity of AN01 and AN03 for α7β1 or αVβ6, respectively, showing that these antibodies are highly selective across the integrin family (Fig. 1E). The observation that AN01 bound to α7β1, but not other β1-subunit-containing heterodimers (e.g. α1β1, α2β1, etc.) confirms that AN01 is specific for the α7 subunit, or an α7β1 complex epitope. Likewise, that AN03 bound to αVβ6, and not to other αV-subunit-containing heterodimers (e.g. αVβ1, αVβ5, etc.), confirms that AN03 is specific for the β6 subunit, or potentially an αVβ6 complex epitope. For α7 or β6 to be expressed at the cell surface, they must be paired with β1 or αV, respectively. Consistent with this, our IP-MS experiments showed peptides from α and β subunits for both targets. Together, these findings support the IP-MS results described above and confirm binding of AN01 to the α7β1 integrin heterodimer, AN02 to HLA-A1, and AN03 to integrin αVβ6 heterodimer.

### Integrin α7 and integrin β6 antibodies enrich for CRC CICs

**Figure 2:**
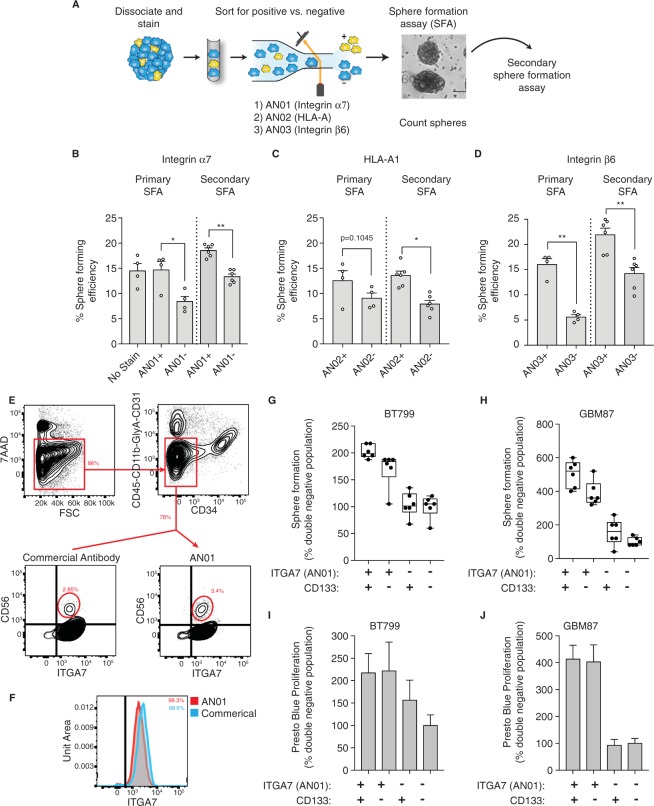
AN01 and AN03 enrich for self-renewing CRC CICs, AN01 is a GBM and muscle stem cell marker. (**A**) Schematic of FACS approach. POP92 spheres were dissociated, stained with indicated antibody and viability dye, and top and bottom 10% of expressing cells were sorted into sphere formation assays (SFAs). Primary sphere formation was assessed by sphere counting. Primary spheres were dissociated, cells were counted and equal numbers were seeded into secondary SFAs. (**B**) Bar charts showing the mean sphere forming efficiency (SFE) as % of cells giving rise to a sphere, for FACS sorted high and low staining AN01 (integrin α7) and (**C**) AN02 (HLA-A1) and **(D)** AN03 (integrin β6), with individual biological replicates indicated as unfilled circles. The primary SFA is shown to the left of the dotted vertical line, and the secondary SFA is shown to the right. Error bars represent SEM, and significance was calculated by paired, two tailed t-test. (**E**) Human muscle stem cells are readily detected by FACS. Biopsy sample is initially gated for live (7−AAD^negative^ left), CD45, CD31, Cd11b, Glycophorin A, CD31, and CD34 all negative (top). True human muscle stem cells are detected by selecting CD56 and integrin α7 double positive cells with both AN01 (right) and the commercial antibody (left). (**F**) Histograms of cells stained with AN01 and commercial antibody reveal the same percentage of integrin α7 positive cells after gating for live and lineage negative cells. (**G**,**H**) Box and whisker plots showing relative sphere formation for **(G)** human, patient-derived GBM cell lines BT799 and (**H**) GBM87, FACS sorted into four quadrants based on AN01 (integrin α7) and CD133 expression. Sorted populations were: α7+/CD133^high^, α7+/CD133^low^, α7−/CD133^high^ and α7−/CD133^low^. Sphere formation is represented for each sorted quadrant as % sphere formation relative to the mean α7−/AC133^low^ (double negative) control. Individual replicates are illustrated as filled circles, the box represents standard deviation of the mean. Results shown are from one representative experiment. N = 2 biological replicates. (**I**) Presto blue measure of cell viability from BT799 SFA (see (G)) and (**J)** GBM87 SFA (see(H)). Error bars represent standard deviation from 6 replicates from a single representative experiment. (N = 2). *p < 0.05, **p < 0.01, ***p < 0.001.

Non-adherent sphere formation is a well-established *in vitro* surrogate assay for CIC function and self-renewal^25^. To test the abilities of our antibodies to prospectively identify CRC CICs, we stained POP92 cells independently with each antibody, sorted by FACS for the high and low staining cells, and performed *in vitro* sphere formation assays (SFAs) with each population (Fig. 2A). AN01 and AN03 were each able to significantly enrich for sphere forming cells (Fig. 2B–D) while AN02 showed a trend towards enrichment but did not achieve statistical significance (Fig. 2C). To test for functional self-renewal capacity, the spheres were dissociated, counted and serially plated into secondary SFAs. The increases in sphere forming efficiency (SFE) were maintained on secondary passage for populations enriched with each of the antibodies, indicating that these antibodies may enrich for self-renewing, stem-like CICs in the POP92 model, *in vitro* (Fig. 2B–D). Together, these findings validate our CellectAb approach for identification of antibodies specific for a cell subpopulation of interest. Moreover, these antibodies can be readily used to identify the protein antigens, and used to enrich for a functionally relevant cell population.

### Integrin α7 antibody enriches for other types of stem cells

While it has not been previously implicated in CRC, the laminin receptor integrin α7 has been described as a human muscle stem cell marker^33^. We used our anti-integrin α7 antibody on a human muscle biopsy to isolate human muscle stem cells, and found that it was of comparable effectiveness as a commercially available antibody (Fig. 2E,F). Given that CD133 is an established marker in GBM, that growth on laminin supports GBM and other types of stem cells, and that the related integrin α6 marks GBM stem cells^34–36^, we hypothesized that the closely related integrin α7 may also play a role in GBM. We used two patient-derived GBM models, which maintain the heterogeneity of the patient tumor, and tested whether the combination of CD133 and integrin α7 antibodies could be used to isolate GBM CICs. GBM spheres were dissociated, co-stained for CD133 and integrin α7 and sorted into four quadrants; CD133^high^/α7+, CD133^low^/α7+, CD133^high^/α7− and CD133^low^/α7−. Sphere formation and proliferation were assessed. In both models, the double negative cells (CD133^low^/α7−) formed the least spheres and, as expected, the double positive population (CD133^high^/α7+) formed significantly more spheres (Fig. 2G–J). Interestingly, in both models, the integrin α7 single positive (CD133^low^/α7+) cells formed more spheres and exhibited more proliferation than the single positive CD133 cells (CD133^high^/α7−), suggesting that integrin α7 may be a bonafide GBM stem cell marker with potentially greater utility than even CD133 (Fig. 2G–J). Importantly, Haas and colleagues also recently identified integrin α7 as a functional marker in GBM^37^, further supporting to our observations.

### Integrin β6 is required for sphere formation

**Figure 3:**
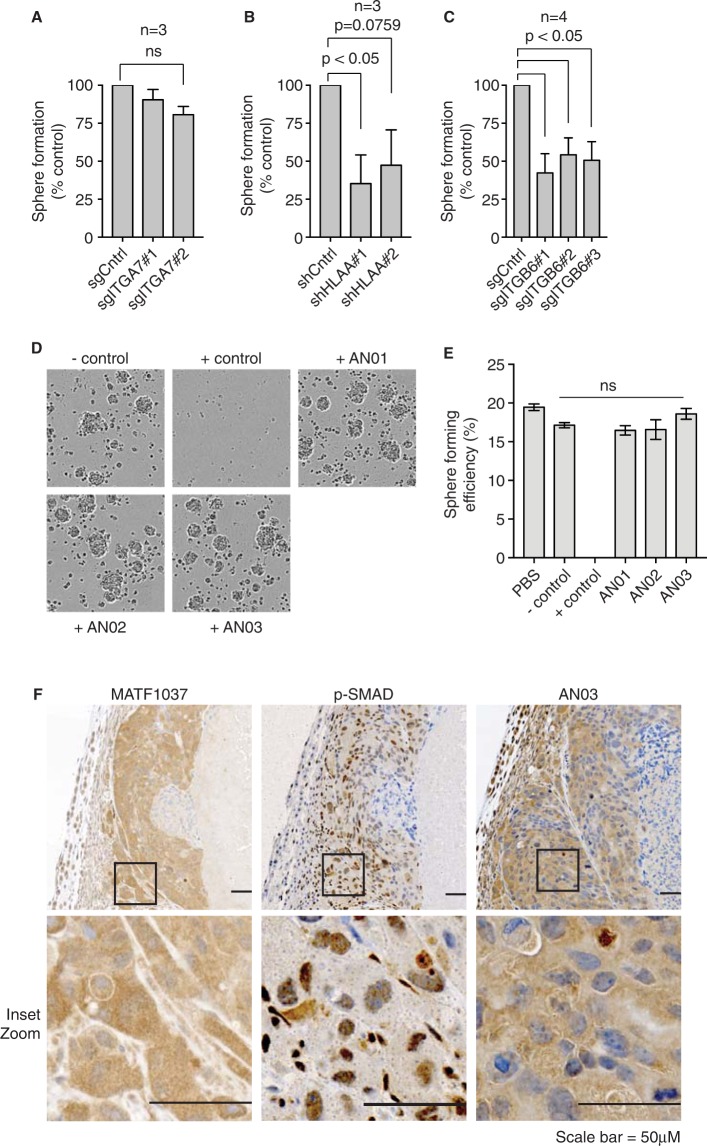
Integrin β6 is required for sphere formation. (**A**) Bar plot illustrating mean sphere forming efficiency (SFE) for FACS sorted AN01 negative POP92 cells stably transduced with unique gRNAs against ITGA7 (sgITGA7s) or a control guide (sgLacZ). N = 3 biological replicates. (**B**) Mean SFE for FACS sorted AN02 negative POP92 cells stably transduced with shHLA-A. N = 3. (**C**) Mean SFE for FACS sorted AN03 negative POP92 cells stably transduced with sgITGB6. N = 4. All error bars represent SEM and significance was calculated by paired student’s t-test. (**D**) Pictures of POP92 cells from growth assays in the presence of 100 μg/mL AN01, AN02, or AN03 antibodies. The negative control was a non-binding IgG raised against maltose binding protein. The positive control was an anti-ROBO4 IgG previously found to block growth of POP92 cells. (**E**) Barplots showing the results of POP92 sphere forming assays in the presence of the indicated antibodies. Controls were the same as described in part D. PBS = phosphate buffered saline. Error bars represent standard deviation (n = 3) and ‘ns’ is not significant. (**F**) POP92 xenograft tumour sections stained by immunohistochemistry (IHC) with MATF1037 (anti-ITGB6 antibody), phospho-SMAD3 S424 + S425 antibody or AN03. Scale bar is 50 uM. *p < 0.05, **p < 0.01, ***p < 0.001.

We hypothesized that the antigens for our antibodies may impact on the capability of CICs in sphere formation and self-renewal. To test this, we used the CRISPR-Cas9 system to knock out *ITGA7* or *ITGB6* in POP92 cells and assessed sphere formation. Cells were separately treated with different short guide RNAs (sgRNA) targeting *ITGA7* or *ITGB6* to generate knockout populations. At this time, sgITGA7 cells were stained with AN01 and sgITGB6 cells were stained with AN03, and the presumptive knockout populations (α7KO and β6KO, respectively) were FACS sorted into sphere formation assays. We observed no significant effect of integrin α7KO on POP92 sphere formation with two validated sgRNAs compared to sorted control cells, indicating that this integrin is not required for *in vitro* sphere formation in our CRC CICs (Fig. 3A). In contrast, β6KO cells were severely impaired in sphere formation (Fig. 3C). We also used shRNAs against HLA-A and FACS sorted for HLA-A low cells and observed a significant reduction in sphere formation (Fig. 3B). We did not observe any effects on POP92 cell proliferation or sphere formation following prolonged treatment of cells with 100 μg/mL AN01, AN02 or AN03 antibodies (Fig. 3D,E). These findings suggest that while integrin α7 marks a functional subpopulation of cells, it does not play a major role in mediating sphere formation or self-renewal in the POP92 model, likely due to functional redundancy with other laminin receptor integrins, whereas HLA-A1 and integrin β6 may mark self-renewing cells and play a functional role in *in vitro* sphere formation.

### AN01 and AN03 antibodies are species cross-reactive

To test if our integrin antibodies are capable of recognizing orthologs of integrins α7 and β6 from other species, we tested binding on mouse cell populations. AN01 was able to bind to mouse splenocytes and infiltrating mouse stromal cells within the POP92 xenograft tumor (Fig. S3A,B). We note that there was a drastic reduction in % integrin α7 positive POP92 xenograft cells compared to POP92 spheres *in vitro* (Figs 1B and S3B). Given that integrin α7 is low in xenograft cells, but is high on mouse cancer-associated stromal cells, it is possible that ITGA7 plays a complex role mediating xenograft-microenvironment interactions. Conversely, AN03 was unable to bind to mouse splenocytes or mouse cells infiltrating the tumor (not shown). It is unclear whether AN03 is unable to bind mouse integrin β6 or if it is not expressed on these cell types. Given the finding that integrin β6 may be a potential therapeutic target, we assessed our antibody for binding to the *Macaca fascicularis* (cynomolgus monkey) integrin αVβ6 heterodimer overexpressed in CHO cells. *M. fascicularis* is used extensively in toxicology studies experiments because of its close physiology to humans. Importantly, AN03 is cross-reactive with *M. fascicularis* integrin β6 (Fig. S3C), demonstrating that our cell-based selection method can quickly and robustly generate highly specific and species cross-reactive antibodies that could be tested further in preclinical models, including murine and non-human primate.

### AN01 and AN03 antibodies bind structural epitopes

In order to investigate the epitope specificity and utility of our antibodies in standard biochemical assays, we tested them in various applications including ELISAs and western blots. AN01 bound specifically, albeit weakly, to recombinant mouse integrin α7β1 in one experiment (Fig. S3D). In contrast, AN03 could not recognize its recombinant protein target *in vitro* (data not shown), suggesting that it binds a conformation-specific structural epitope that requires the heterodimer to be properly displayed in the plasma membrane. Additionally, none of the three antibodies were functional in a denaturing western blot (data not shown), again suggesting that they do not bind to linear, but rather structural epitopes. We also optimized and tested AN03 by immunohistochemistry (IHC) on paraffin-embedded xenograft samples. Our antibody performed comparably to a commercial anti-integrin β6 antibody, and was highly specific for the integrin β6-positive POP92 tumour, compared to an integrin β6-negative breast cancer xenograft (Fig. S3E).

Integrin αVβ6 acts as a receptor for latency-associated peptide (LAP) associated with TGFβ, thus freeing the active form of TGFβ and promoting downstream signaling. To test whether integrin αVβ6 is associated with TGFβ signaling in POP92 xenografts, we used phospho-SMAD3 as a read out of TGFβ activation. We stained sequential tumor sections by IHC with a commercial anti-integrin β6 antibody, AN03 and phosho-SMAD3. We observed that areas in the tumor with high nuclear localized phospho-SMAD3 (i.e. high TGFβ activation) also expressed integrin β6, with either antibody (Fig. 3F). These observations suggest a potential role for integrin αVβ6 in mediating CRC CIC function through TGFβ signaling.

### AN01 target validation identifies protein-protein interactions

In addition to integrin α7β1, we observed additional peptides by IP-MS with our AN01 Fab across multiple cell lines (Table S2), which correspond to the heavy (CD98hc) and light (CD98lc) chains, respectively. CD98 is a multifunctional heterodimeric cell-surface amino acid transporter previously associated with cancer initiating populations^38^. We ruled out direct binding of AN01 to CD98 by ELISA, shRNA knockdown and CD98 overexpression (Fig. S3F) and data not shown) and confirmed endogenous co-IP of CD98 with integrin α7β1 by western blot in multiple cell lines (Fig. S3G). The CD98lc (SLC7A5 also known as large amino acid transporter, LAT1), is known to interact with, and play a role in activation and clustering of, β1 integrins^39^. Our data confirms this interaction in CICs, specifically with the α7β1 heterodimer. Together, these findings demonstrate the utility of our methodology at identifying not only novel cell surface targets, but also some of their interacting partners.

## Discussion

Our cell-based antibody identification method (i.e. CellectAb) represents a powerful new approach for discovery of novel cell population-specific antibodies and corresponding targets. While hybridoma technology has been traditionally used to discover antibodies, our approach has several distinct advantages. First, it is rapid and cost effective; within a week antibodies are generated against cells of interest and antibody production can be scaled up within a month. This reduces the time and work load significantly compared to a 30 week timeline from immunization to scaled production from a hybridoma^1^. Second, the recombinant approach yields a sequenced and modular antibody fragment. This allows for simplified cloning and expression as multiple modalities including Fab, IgG and bi-specifics (such as bispecific T-cell engagers (BiTEs))^40^ or chimeric antigen receptor (CAR) T-cells^41^. Third, as the fixed framework is fully human, no humanization is required. Finally, *in vitro* selections with synthetic repertories allow the production of species cross-reactive antibodies, which are highly desirable for many reasons, including the enabling of reliable translational medicine toxicology studies in model organisms. In contrast, generation of species cross reactive antibodies by animal immunization for highly conserved epitopes can be challenging due to self-tolerance mechanisms that often delete cross reactive antibodies. Our fully synthetic approach is not limited by any clonal deletion and could be used to target any antigen, including highly conserved functional epitopes.

Integrin α7 has not previously been implicated in CRC CICs. In our model, integrin α7 was co-expressed with CD133 on the CIC fraction and it enriched for sphere forming cells, but integrin α7 was not required for sphere formation or proliferation under the conditions tested. As a cell adhesion molecule and laminin receptor, integrin α7 may be dispensable *in vitro*, but may play a greater role in the tumor microenvironment of a xenograft or patient. Indeed, while integrin α7 levels are greatly reduced on bulk tumour *in vivo*, we have not ruled out the expression of integrin α7 on rare CICs. The high level of integrin α7 expression on supporting mouse stromal cells may also contribute to bulk tumour growth or CIC function. It also seems likely that other laminin-binding integrins could be functionally redundant in CRC and that targeting multiple such integrins could be required to see functional effects in the POP92 model. Our somewhat conflicting results are perhaps not surprising, as the role of integrin α7 is disputed in other cancer models, with certain studies implicating *ITGA7* in a tumor suppressor role^42^. The role of α7 will need to be further investigated in other CRC models in appears to be highly context-dependent. For example, the role of integrin α7 is becoming more clear in GBM. For *in vitro* culture, embryonic stem cells and GBM cells are cultured on laminin to maintain a pluripotent state^35,36^ and integrin α6, the closest homolog of α7, also binds laminin and has been described as a GBM CSC marker^34^. Since initiation of this study, another group described integrin α7 as a functional marker in GBM and proposed it as a potential therapeutic target^37^.

Integrin β6 has been previously implicated in the invasion and metastasis of many tumours including colon and breast cancers^43,44^, but has only been linked directly to CICs in squamous carcinoma^45^. In our model, integrin β6 was co-expressed with CD133 and could be used to prospectively isolate CRC CICs. Furthermore, perturbing integrin β blocked sphere formation, indicating that this model requires the αVβ6 to grow as self-renewing tumor spheroids. At no point did we observed the formation of organoids. The αVβ6 heterodimer binds to an RGD motif in ligands including fibronectin and vitronectin, and the principal ligand the latency-associated protein of TGFβ (LAP-TGFβ). Binding of integrin αVβ6 to LAP induces a conformational change that releases activated TGFβ, which in turn can activate TGFβ signaling^30^. The link between integrin β6 and CRC CICs, described above, may be mediated by one or more of these ligands. IHC staining showed co-localization of integrin β6 and phospho-SMAD3, a readout of TGFβ activation^46^. This suggests that the effects of integrin αVβ6 in CRC CICs may be mediated through the TGFβ pathway, although further investigations are required to confirm this hypothesis. Integrin β6 is up-regulated in tissues undergoing development, wound healing, and in malignant cancers especially those with metastasis^43,44^. However, unlike other integrin subunits, integrin β6 has restricted expression with low to undetectable levels in most healthy tissues^47^, making it a promising cancer-associated antigen or direct therapeutic target. While AN03 does not block the function of integrin β6 or show anti-proliferative effects *in vitro*, it may be a promising agent to target CRC CICs with antibody-drug conjugates or with bispecific T-cell engagers (BiTEs) or CAR T-cells that engage the immune system. Indeed, our results support the idea that integrin β6 is a viable target for immunotherapy^48,49^.

The HLA proteins, including HLA-A1 are responsible for the regulation of the immune system in humans. That reduction of HLA-A reduced *in vitro* sphere formation suggests that HLA-A may also play an unknown functional role in CIC function, independent of immune interactions. It is plausible that the overexpression of this, or other MHC, may be reflective of an immunostimulatory state, that could perhaps be exploited with immunotherapy.

The development and application of our CellectAb methodology has yielded three new affinity reagents, one specific for HLA-A1 haplotype, and two against distinct integrin subunits, α7 and β6. Despite the sequence homology between various integrin subunits, our antibodies show exquisite specificity and no cross reactivity to other α or β subunit isoforms. Moreover, AN02 binds HLA-A1 and not other HLA-A serotypes. Taken together, these results clearly validate that the CellectAb approach coupled with a phage-displayed Fab library of restricted diversity in only two CDRs (L3 and H3) is capable of generating highly selective antibodies for functional subpopulations of cells. A wide variety of antibodies against the surface of cells have been used to identify, study and target clinically relevant subpopulations in cancer. Historical approaches utilizing animal immunization have been highly effective at identifying new targets, but are time consuming, low throughput, and yield animal antibodies that cannot be directly transplanted into human patients. Our CellectAb methodology can be readily applied to other cell subpopulations to facilitate the rapid identification of novel targets and simultaneous generation of potent and specific antibodies.

## Methods

### Cell culture and cell lines

MAC-LS-2, BT549, MCF7, HCC1954, BT20, HCT116, HPAC, PL45, LNPAC, PC3, DU45, HUVEC, HEK293T and Caco2 cells were obtained from the American Tissue Type Collection (ATCC) and grown under suggested conditions. KP2 and KP4 were obtained from Japan Health Sciences Foundation (JHSF) and grown under suggested conditions. HAP1 cells were obtained from Horizon Genomics and grown under the suggested conditions. HEK293T-CD133-GFP cells were generated by transducing HEK293T cells with a lentiviral-based CD133-GFP fusion construct. GBM87 (MGG87) cells were a kind gif of Dr. Hiroaki Wakimoto (Massachusetts General Hospital, Boston MA, USA). BT799 cells were derived in the lab of Sheila Singh (McMaster University, Hamilton, Ontario, Canada) from a primary glioblastoma.

### POP92 cell culture

The POP92 colorectal cancer spheroid line was originally derived from a stage IV sigmoidal colon adenocarcinoma from a 45-year-old female patient donor xenografted in an NSG mouse. After xenograft harvest and dissociation, POP92 cells were maintained *in vitro* in suspension flasks (Starstedt, Cat# 83.3911.502) in serum free media, supplemented with EGF and bFGF as previously described^25^. For expansion, POP92 spheres were passaged every four to seven days. In short, spheres were harvested by gentle centrifugation (200 g), trypsinized with 0.25% Trypsin-EDTA (ThermoFisher, Cat# 25200056) for 5 minutes at 37 C. Trypsin was washed out with serum-free DMEM/F12 (ThermoFisher, Cat# 11320) and cells were triturated at least 30 times against the bottom of the tube with a 5 mL pipette to achieve a single cell suspension. Any remaining clumps of cells were removed with a 40 uM cell strainer (Falcon, Cat# 352340). Cells were counted with a Z2 Coulter Counter (Beckman Coulter, Cat# 6605700) and re-seeded into freshly prepared media. All additional adherent cell lines (see Supplemental Methods) were maintained in the ATCC recommended media supplemented with 10% FBS and 1% penicillin/streptomycin in tissue culture treated vessels and passaged every 2–4 days by trypsinization. Cell lines were tested regularly for mycoplasma.

### Isolation of AC133+ CIC-enriched fraction

POP92 has been previously validated to contain a population of AC133 positive CICs^25^. To isolate the AC133 positive CIC population, POP92 spheroids were mechanically and enzymatically dissociated with 0.25% trypsin, washed well and strained to single cell suspension, then stained with AC133 conjugated to Alexa-488 fluorophore (Miltenyi Cat# 130-105-225) and 7-AAD viability dye (Biolegend Cat# 420404). Live, singlet cells were FACS sorted using a BD FACSAria into the top 10% of AC133^high^ cells (CIC-enriched fraction), and the bottom 20% AC133^low^ cells (low CIC fraction).

### Antibody Selection Strategy

A library of highly diversified (>10^11^) human Fab fragments expressed on M13 bacteriophage was created and prepared as described^50^. Naïve library was pre-absorbed on AC133^low^ cells to remove non-specific binders. Unbound Fab-phage were incubated with the AC133^high^ CIC population. The cell pellet was washed, and bound phage were eluted and amplified in an *E. coli* host. This selection process was repeated for a total of 4 rounds. In parallel, non-specific binders were amplified through 4 rounds of selection by direct incubation with AC133^low^ cells, followed by elution and amplification. After the selection process, the DNA sequences encoding for the CDRs of Fab-phage output from round 4 were subjected to PCR amplification and deep sequencing. Sequences were processed as in^50^, and top hits were selected based on high read counts in positive pool and low or no read counts in the negative selection pool. The top candidate hit sequences were cloned into IPTG-inducible expression vectors, and Fab proteins were produced and purified from BL21 *E. coli*, as described^26^.

### Flow cytometry

POP92 cells were processed as above in “Isolation of AC133^high^ CIC-enriched fraction”. All adherent cell lines were harvested by aspirating media, washing in PBS then fully dissociated in sterile non-enzymatic dissociation buffer, DB (1 mM EDTA, 137 mM NaCl, 6.7 mM NaHCO_3_, 5 mM KCl and 5 mM D-Glucose) for 5–10 minutes. Samples were then diluted with PBS 2% FBS, filtered through 40μM mesh and stained with appropriate antibody dilution and viability dye. Data acquisition was performed on a BD LSR Fortessa X20 (BD Biosciences) and FACS sorting was performed using a BD FACS Aria or BD FACS Influx.

### Integrin overexpression

HEK293T cells were seeded at 25,000 cells per well into 12 well plates. In parallel, a large batch of control cells were seeded into a 15 cm plate. After 24 hours, 12 well plates were co-transfected with indicated integrin pairs (see Table of overexpression plasmids in Supplemental Methods) using XtremeGene9 (Roche, Cat# 63650809001) to the manufacturer specifications. 48 hours after transfection, cells were dissociated with non-enzymatic dissociation buffer (DB) containing 1 mM EDTA, 137 mM NaCl, 6.7 mM NaHCO_3_, 5 mM KCl and 5 mM D-Glucose for 5–10 minutes at room temperature. Un-transfected in-tube control cells were incubated with 1:15,000 dilution of Carboxyfluorescein succinimidyl ester (CFSE) CellTrace dye (ThermoFisher, Cat.# C34570) for 30 minutes at 37 C. Excess CFSE was washed away with PBS containing 2% FBS, controls cells were dissociated with DB as above. Each transfection was split into two tubes, and CFSE-stained untransfected control was added in equal amounts to all tubes. For each transfection, one tube was stained with 2ug/ml AN01 scIgG and the other with 2ug/ml AN03 scIgG, followed by anti-human APC secondary (Jackson, Cat#109-136-097) and DAPI viability dye. Data was acquired on a BD LSR Fortessa X-20. Cells were gated for live singlets, and segregated on the basis of CFSE staining for control and query cells. The mean APC fluorescent intensity was calculated for both the query and control cells in each tube. The difference between these measures was calculated as a % maximum fold change. This experiment was performed in biological triplicate.

### Immunoprecipitation (IP)

Cells were washed 2x in ice cold PBS, then lysed in lysis buffer (10% Glycerol, 50 mM HEPES-KOH pH 8.0, 100 mM KCl, 2 mM EDTA, 0.1% NP-40, 1x Protease inhibitor (Sigma #), 10 mM NaF, 0.25 mM NaOVO_3_, 5 nM Okadaic acid, 5 nM Calyculin A, 50 mM β−glycerolphosphate) for 60 minutes with intermittent vortexing. Lysates were freeze-thawed at −80C, thawed on ice, then centrifuged at >15,000 RPM for 60 minutes. Each supernatant was transferred to a new tube, and spun >15,000 RPM for additional 30 minutes. Protein supernatants were incubated with 10μg of antibody overnight at 4 C on a nutator. Immunocomplexes were precipitated with Protein G (for IgGs), FLAG-M2 (FLAG-tagged Fabs) or streptavidin beads (Avi-tagged Fabs), as indicated (beads purchased from Pierce, Cat# 53125, Sigma, Cat# A2220 and Pierce, Cat# 20347, respectively). Beads were washed well with lysis buffer, followed by H_2_O. Proteins were eluted in SDS-PAGE sample buffer (NuPage gel system, ThermoFisher, Cat# NP0301) for western blot, or processed as below in 2.4.8 for mass spectrometry (MS).

### Mass Spectrometry (MS)

Bead bound proteins for MS were eluted in 0.15% trifluoroacetic acid (TFA), then adjusted to ~pH 8.0 with 1 M NH_4_HCO_3_. Samples were reduced with 1/10^th^ volume 45 mM DTT at 60 C for 20 minutes. Samples were then cooled to room temperature and incubated with cysteine blocking reagent (BioShop Cat# IOD500.5) for 15 minutes in the dark. Peptides were digested with Biolab modified trypsin (TPCK Cat# P8101S) at room temperature overnight. Each sample was re-acidified to 1% TFA and purified using C18 stageTip (Thermo Scientific, Cat# SP301) according to the manufacturer’s specifications, dried using a speed vacuum, and analyzed on a mass spectrometer.

### RNA interference (RNAi) experiments

pLKO vector containing indicated shRNA (see Table of RNAi reagents in Supplemental Methods) were packaged into lentiviral particles. Cells were infected at MOI <1 and stably transduced cells were selected in 2 μg/mL puromycin.

### CRISPR/Cas9 single gene knockout generation

Indicated sgRNAs (see Table of CRISPR reagents in Supplemental Methods) were cloned into Cas9-containing vector, LentiCRISPR v2 (Addgene Cat#52961). The resulting vectors were packaged into lentiviral particles and cells stably transduced by selection in 2μg/ml puromycin for 48 hours, allowed to recover for 24 hours, then knockout cells were FACS sorted.

### Isolation of human skeletal muscle samples

Human skeletal muscle specimens were obtained from otherwise healthy patients undergoing elective spinal surgery at Toronto’s St. Michael’s hospital. Patients provided informed consent prior to collection of the skeletal muscle sample. Use of human tissue samples was approved by the University of Toronto Research Ethics Boards (REB# 13–370) and all experiments were performed in accordance with relevant guidelines and regulations.

### Human muscle stem cell identification

We assessed in parallel the ability of commercially available ITGA7 antibody and AN01 to detect human muscle stem cells by flow cytometry using the method described in^33^. Briefly, human skeletal muscle was finely minced and digested in 630 U/ml collagenase type 1A (Sigma, Cat# C9891) on a rocking shaker for 1 hour. Dispase II (Life Technologies, Cat# 17105-041) was added to the digesting tissue at 1.1 U/ml for 1 hour. Digested tissue was filtered using a 70 um (Miltenyi Biotec, Cat# 130-11-916) and 40 um (Fisher Scientific, Cat# 08-771-1) cell strainer and centrifuged at 400 g for 15 minutes. Cells were resuspended in buffer (2.5% goat serum and 2 mM EDTA in PBS) and conjugated or primary antibodies added to the cell suspension and incubated at 4 °C for 1 hour. Antibodies and dilutions were as follows: CD45-PE (1:25, eBioscience, Cat# 12-0459-42), CD31-PE (1:10, Abcam, Cat# ab27334), CD11b-PE (1:25, eBioscience, Cat# 12-0118-42), Glycophorin A-PE (1:50, Abcam, Cat# ab26016), CD34-PE-Cy7 (1:40, BD, Cat# 560710), CD56-APC (1:20, BD, Cat# 557711), ITGA7-FITC (1:10, Novus Biologicals, Cat# NBP1-54412), AN01 (50 µg/ml). Stained cells were centrifuged at 400 g for 15 minutes. For samples stained with AN01, cells were resuspended in buffer with goat anti-mouse AlexaFluor488 secondary antibody (1:500, Life Technologies, Cat# A-11001) at 4 °C for 30 minutes, then centrifuged for 15 minutes at 400 g. Finally, 7-Aminoactinomycin D (1:1000, Life Technologies, Cat# A1310) was added and samples were immediately run on the BD Canto flow cytometer. Human muscle stem cells were gated as follows: 7AAD^−^/CD31^−^/CD45^−^/CD11b^−^/GlycA^−^/CD34^−^/CD56^+^/ITGA7^+^.

### GBM self-renewal and proliferation assays

Glioblastoma cells were cultured at 37 °C and 5% CO_2_, as neurospheres in suspension using serum-free human neural stem cell NeuroCult™ basal media (STEMCELL Technologies, Cat# 05750) supplemented with Neurocult NS-A proliferation supplements (STEMCELL, Cat# 05753), 2 μg/ml heparin (STEMCELL, Cat# 07980), 20 ng/mL recombinant human EGF (STEMCELL, Cat# 02633), and 10 ng/ml recombinant human FGF (STEMCELL, Cat# 02634). The neurospheres were enzymatically dissociated into a single cell suspensions using Liberase™ TM (Roche). The cells were stained at 4 °C for CD133 using a monoclonal CD133 antibody conjugated to PE (Miltenyi Biotec, Cat# 130-090-853), and for ITGA7 using AN01 along with a goat anti-human IgG allophycocyanin (APC) AffiniPure F(ab’)_2_ fragment secondary antibody (Jackson ImmunoResearch, Cat# 109-136-097) before being sorted by FACS using a MoFlo XDP (Beckman Coulter) into four populations: CD133+/ITGA7+, CD133+/ITGA7−, CD133−/ITGA7+, and CD133−/ITGA7−. The proliferation assay is performed by sorting 1000 cells per well, per population, into a tissue culture treated 96-well plate (Falcon). The PrestoBlue® cell viability reagent (Invitrogen, Cat# A13262) is then added at 10% (v/v) after two weeks and read using the FLUOstar Omega spectrometer (BMG Labtech). Similarly, self-renewal assays are performed by sorting 200 cells per well, per population into a 96-well plate (Falcon). The plate is incubated for two weeks before the number of neurospheres were counted per well. In these experiments, a neurosphere was defined as a spherical cluster of 4 cells or more.

### Immunohistochemistry

Tumours were surgically isolated and immediately immersed in 10% formalin (Sigma Cat# HT501128) for at least 48 hours before being transferred into 70% Ethanol. Tissue was dehydrated sequentially in increasing concentrations of ethanol, and then xylene. They were then embedded in paraffin, sectioned and placed on slides. Sections were de-parafinized and rehydrated in citrate buffer pH = 6 (for ITGB6 antibodies, AN03 and mouse monoclonal MATF1037, Monash Antibody Technologies Facility, Monash University, Australia) or Tris-EDTA pH = 9 (for anti-phospho-Smad3 S424 + S425, Abcam, Cat# Ab52903) for antigen retrieval. Primary antibody MATF1037 was used at a dilution of 1:10,000 for one hour, AN03 was used at 15 ug/ml and phospho-Smad3 at 1:50 overnight. Staining was detected with species-specific secondary antibodies conjugated to HRP using the IHC DAB polymer detection kit. Slides were counterstained with hematoxylin, imaged on the Zeiss Axioscan slide scanner, and analyzed in Zen Blue.

## Supplementary information

Supplementary Information
