# Supplementary material for: A rapid in vitro methodology for simultaneous target discovery and antibody generation against functional cell subpopulations
Source: Sci Rep. 2019 Jan 29;9:842. doi: 10.1038/s41598-018-37462-1 (PMC6351593; doi:10.1038/s41598-018-37462-1)
Supplement: Supplementary file 1 — Supplementary Information [file 41598_2018_37462_MOESM1_ESM.pdf]

Supplemental material for:

A rapid *in vitro* methodology for simultaneous target discovery and antibody generation against functional cell populations

Author List:

Allison M.L. Nixon[1,2], Alejandro Duque[1], Nicholas Yelle[3,4], Megan McLaughlin[1,2], Sadegh Davoudi[1,5], Nicolas M. Pedley[6], Jennifer Haynes[6], Kevin R. Brown[1], James Pan[1], Traver Hart[1,a], Penney M. Gilbert[1,5], Sheila K. Singh[3,7], Catherine A. O'Brien[6,8,9], Sachdev S. Sidhu[1,2], and Jason Moffat[1,2,10*]

Author Affiliations:

[1]Donnelly Centre, University of Toronto, Toronto, Canada, M5S3E1
[2]Department of Molecular Genetics, University of Toronto, Toronto, Canada, M5S1A1
[3]McMaster Stem Cell and Cancer Research Institute, McMaster University, Hamilton, Canada, L8S 4K1
[4]Department of Biochemistry and Biomedical Sciences, McMaster University, Hamilton, Canada, L8N 3Z5
[5]Institute of Biomaterials and Biomedical Engineering, University of Toronto, Toronto, Canada, M5S 3G9
[6]Princess Margaret Cancer Centre, University Health Network, Toronto, Canada, M5G2M9
[7]Faculty of Health Sciences, Department of Surgery, McMaster University, Hamilton, Canada, L8N 3Z5
[8]Department of Surgery, Toronto General Hospital, Toronto, Canada, M5G2C4
[9]Department of Laboratory Medicine and Pathobiology, University of Toronto, Toronto, Canada, M5S1A1
[10]Canadian Institute for Advanced Research, Toronto, Canada, M5G1Z8
[a]Current address: Department of Bioinformatics and Computational Biology, MD Anderson Cancer Center, Houston, Texas, USA, 77030

Corresponding Author:

Dr. Jason Moffat, Professor, Donnelly Centre for Cellular and Biomolecular Research, University of Toronto, Room 802- 160 College Street. Toronto, Ontario, Canada, M5S 3E1. Email: j.moffat@utoronto.ca

Supplemental Methods:

Phage-Fab Library design and construction.

Using a synthetic antibody library with equivalent sequence diversity incorporated in both complementarity-determining regions (CDRs) H3 and L3 (library F), it has previously been shown that CDR-L3 is equally as capable of contributing to functional antigen-binding sites as is CDR-H3, a notion that contradicts studies of natural antibodies that have shown a dominant role for CDR-H3 in antigen binding (12). Library F contains diversity in four CDRs (H1,H2,H3, and L3); however, in the majority of functional Fabs isolated from library F, diversity in CDR-H1 and CDR-H2 is modest, and it is the contributions of CDRs H3 and L3 that largely dictate binding specificity and affinity (12, 13). To take advantage of these observations and create a workflow highly amenable to deep sequencing coupled with cell-based panning, we constructed a synthetic Fab library (library I) designed to restrict diversity to CDR-L3 and CDR-H3 (Figure S1A-B). Within CDR-H3 and CDR-L3, library I contained a tailored amino acid composition that was more diverse and polar than that of library F to enhance solubility of the binding sites. Diversity was still biased in favour of Tyr (25%), Ser (20%) and Gly (20%), but also contained 5% each of Ala, Phe, Trp, ,His, Pro, Asp and Arg. We also allowed for broad length diversity in CDR-H3 and CDR-L3 by incorporating loop lengths that are found within these regions of natural antibodies (Figure S1C). Library I was constructed using an anti-maltose-binding protein Fab as the template and contained 8 x 10^{10} unique clones. Sequencing of the naïve library revealed the incorporation of diversity in approximately 80% of the population within each CDR and the retention of template sequence in the remainder. Restriction of sequence diversity to the CDR-H3 and CDR-L3 loops provides a fixed backbone that facilitated design of primers for next generation sequencing of multiplexed library pools (Figure S1D).

Integrin knockout cell lines

HAP1 integrin knockout cells were obtained from Horizon Discovery (formerly Haplogen Genomics): *ITGA1* knockout - α1 KO-(Cat# HZGHC001039c001), *ITGA2* knockout- α2 KO-(Cat# HZGHC001073c010), *ITGA3* knockout - α3 KO-(Cat# HZGHC001043c008), *ITGA4* knockout - α4 KO-(Cat# HZGHC001077c009), *ITGA5* knockout - α5 KO-(Cat# HZGHC001085c001), *ITGA6* knockout - α6 KO-(Cat# HZGHC001052c010), *ITGA7* knockout - α7 KO-(Cat# HZGHC001054c012), *ITGA9* knockout - α9 KO-(Cat# HZGHC001050c02), *ITGB1* knockout - β1 KO-(Cat# HZGHC001029c003), *ITGB4* knockout - β4 KO- (Cat# HZGHC001027c011) and *ITGB5* knockout - β5 KO- (Cat# HZGHC001028c001) and maintained in IMDM (ThermoFisher Cat# 12440079) supplemented with 10% foetal bovine serum (FBS) (ThermoFisher Cat# 10437010) and 1% penicillin/streptomycin.

Table of RNAi reagents:

**shRNA ID**	**Target Sequence**	**TRCN Number**
shITGB1#1	GCCTTGCATTACTGCTGATAT	TRCN0000029645
shITGB1#2	GCCCTCCAGATGACATAGAAA	TRCN0000029648
shITGA7#1	GCCCTGGACTATGTGTTAGAT	TRCN0000057709
shITGA7#2	CCTCCGGGATTTGCTACCTTT	TRCN0000057712
shHLA-A#1	CCCTTCCCTTTGTGACTTGAA	TRCN0000057238
shHLA-A#2	CACACCATCCAGATAATGTAT	TRCN0000057239
shITGB6#1	GAAACATTTATGGGCCTTATT	TRCN0000057706
shITGB6#2	GCCAACCCTTGCAGTAGTATT	TRCN0000057707

Table of CRISPR reagents:

**sgRNA ID**	**CRISPR Target sequence**
sgITGA7#1	AGAGTGGACATCGACCAGGG
sgITGA7#3	CAGGGATCGTCCCATGGCCG
sgITGB6#4	ACTGCGGTCTGAGGTGGAAC
sgITGB6#8	AAAGGAACTGCGGTCTGAGG
sgITGB6#9	AAGTGTGTTTGCACAAACCC

Table of overexpression plasmids:

Protein	Origene Catalogue Number
ATP1A1 (control surface protein)	SC119714
ITGA1	SC107298
ITGA1	SC119742
ITGA10	SC117827
ITGA11	SC124146
ITGA2	SC118747
ITGA3	SC303656
ITGA4	SC119587
ITGA5	SC118748
ITGA6 transcript variant 1	SC315867
ITGA6 transcript variant 2	SC120055
ITGA7	SC303148
ITGA8	SC127886
ITGaIIb	SC300066
ITGAL	SC124031
ITGAM	SC315229
ITGAV	SC118750
ITGB1	SC111935
ITGB2	SC320165
ITGB3	SC120057
ITGB5	SC118751
ITGB6	SC128124
ITGB8	SC118752

ELISA

A 384 well immunosorbant plate was coated with purified recombinant protein antigens: ATP1A1 (Origene, Cat.# TP301009), CD98 full length (Abnova, Cat# H00006520-G01), CD98 transcript variant 1 (Origene, discontinued) or mouse integrin $\alpha7\beta1$ (R&D, Cat# 7958-A7) each diluted in PBS overnight at 4C with gentle shaking. The plate was blocked with PBS 0.5% BSA at room temperature for 60 minutes. Block was removed, and antibodies diluted in PBT (PBS with 0.5% BSA and 0.05% Tween-20) incubated in wells for 60 minutes. The primary antibody was washed away at least 4x in PT (PBS 0.05% Tween-20). HRP secondary

antibody was added 1:5000 in PBT for 30 minutes, then washed with PT at least 7x, incubated TMB substrate kit (ThermoFisher, Cat# 34021) and read at 450nm in the Biotek Powerwave XS plate reader.

Western Blotting

Whole cell lysates were prepared using radioimmunoprecipitation assay (RIPA) buffer (ThermoFisher, Cat# 89900) supplemented with HALT protease and phosphatase inhibitor (ThermoFisher, Cat# 78443). Cells were lysed on ice for at least 60 minutes with regular vortexing and pippetting, and the insoluble pellet was spun down at maximum speed for 30 minutes. Soluble proteins were quantified using BCA assay (ThermoFisher, Cat# 23225). Equal protein amounts were mixed with NuPage sample buffer and DTT, boiled for 5 minutes and run on 4-12% gradient Bis-Tris NuPage gels (ThermoFisher, Cat# NP321). Proteins were wet transferred to activated PVDF membranes (GE Healthcare Cat#10600023), and probed overnight at 4C with indicated primary antibodies. The following day membranes were washed in tris-buffered saline with 0.2% Tween (TBST), and incubated with appropriate secondary antibody for 60 minutes at room temperature. Membranes were washed in TBST and developed on the MicroChemi 4.2 (FroggaBio, Cat#95-25-00) using Pierce ECL chemiluminescent substrate (ThermoFisher, Cat#32106). Images were processed in Adobe Photoshop for rotation, cropping, contrast and brightness enhancements. For IP-Western analysis, all input samples were prepared as above, and run in the same way.

Supplemental tables

Table S1: AN01, AN02 and AN03 expression pattern across cancer cells from many tumour types. A panel of cell lines derived from a variety of normal and cancer tissue types were stained were assessed for CD133 expression status and cell surface staining pattern for AN01, AN02 and AN03 antibodies by flow cytometry. Cell lines are classified as either CD133 positive (+) or CD133 negative (-) by transcript and staining with AC133 antibody. Grey shading indicates CD133 positivity. Cell lines are classified for AN01, AN02 and AN03 by percent positive staining for indicated antibody. "+" = 1-25% positive cells, "++" = 25-75% positive cells, and "+++" = 75-100% positive cells. "-" is <1% staining. All percent positives are calculated as % live cells shifting above secondary only control stain.

			Relative Binding		
**Cancer type**	**Cell Line**	**CD133 status**	**AN01**	**AN02**	**AN03**
Breast	MAC-LS-2	+	++	++	+++
	BT549	-	-	-	-
	MCF7	-	++	+	-
	HCC1954	-	-	+	+
	BT20	-	+	-	-
Colon	POP92	+	++	+++	+++
	HCT116	+	++	++	-
	Caco2	+	++	-	-
Pancreas	GP3A	-	-	+++	-
	KP2	-	+	+++	+++
	KP4	-	-	++	-
	HPAC	+	-	-	++
	PL45	+	-	+++	-
Prostate	LnPac	-	++	-	-
	PC3	-	+++	++	-
	DU145	-	-	-	-
Leukemia	HAP1	–	+++	++	-

Non-cancerous	HUVEC	-	+	+++	-
	293T	-	+++	-	-
	293T CD133-GFP	+	+++	-	-

Table S2: IP-MS results 2- AN01 pulls down integrin α7β1 in complex with CD98. Two separate AN01 Fabs (Flag tag IP'd with anti-Flag beads, and biotin tag pulled down with streptavidin beads) were used to IP the target from POP92, Caco-2 colorectal cancer cell line, and MAC-LS-2 a breast cancer cell line. BOC is beads only control. Peptide counts for top validated hits are shown. See Table S3 for full results.

	**Cell line**	**POP92**				**Caco-2**				**Macls2**			
	**IP- Fab-Tag**	**Avi**		**FLAG**		**Avi**		**FLAG**		**Avi**		**FLAG**	
**Target**	**Protein**	**BOC**	**AN01**	**BOC**	**AN01**	**BOC**	**AN01**	**BOC**	**AN01**	**BOC**	**AN01**	**BOC**	**AN01**
α7β1	ITGAB1	0	25	0	19	0	13	0	12	0	0	0	1
	ITGA7	0	28	0	20	0	16	0	9	0	0	0	0
CD98	SLC3A2	2	19	0	12	1	23	0	19	1	19	0	10
	SLC7A5	0	3	0	2	0	3	0	3	0	2	0	3

Supplemental figures

Figure S1

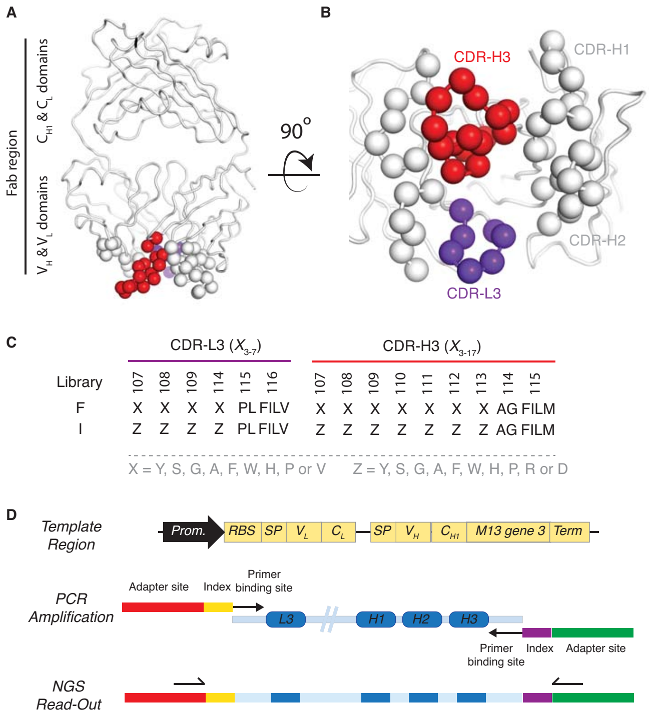

Figure S1*: Library I design features.* The backbones of the heavy- and light-chain variable domains are shown as tubes for the full Fab (A) or the zoom in of the CDRs in the antigen-binding site (B). The frameworks are colored gray and the CDR loops are colored as follows: CDR-L3 (purple), CDR-H1 (grey), CDR-H2 (grey) and CDR-H3 (red). Spheres colored according to the CDR color scheme represent positions that were diversified. The figure was generated using PyMOL with crystal structure coordinates (PDB entry 3PNW). (C) CDR diversity design of libraries F and I. The backbones were fixed according as the parental sequence and at each diversified position, the allowed amino acids are denoted by the single-letter code, where X denotes a mixture of 9 amino acids (Y, S, G, A, F, W, H, P, or V) and Z denotes a mixture of 10 amino acids (Y, S, G, A, F, W, H, P, R, or D) introduced at proportions described in the Methods. The lengths of CDR-L3 and CDR-H3 were varied by replacing the positions denoted by X with 3-7 or 1-17 degenerate codons, respectively. Residue numbering is according to the IGMT scheme. (D) Schematic of the phagemid Fab expression construct and experimental design for amplifying the DNA encoding for CDR-L3 and CDR-H3 for paired-end deep sequencing.

Figure S2

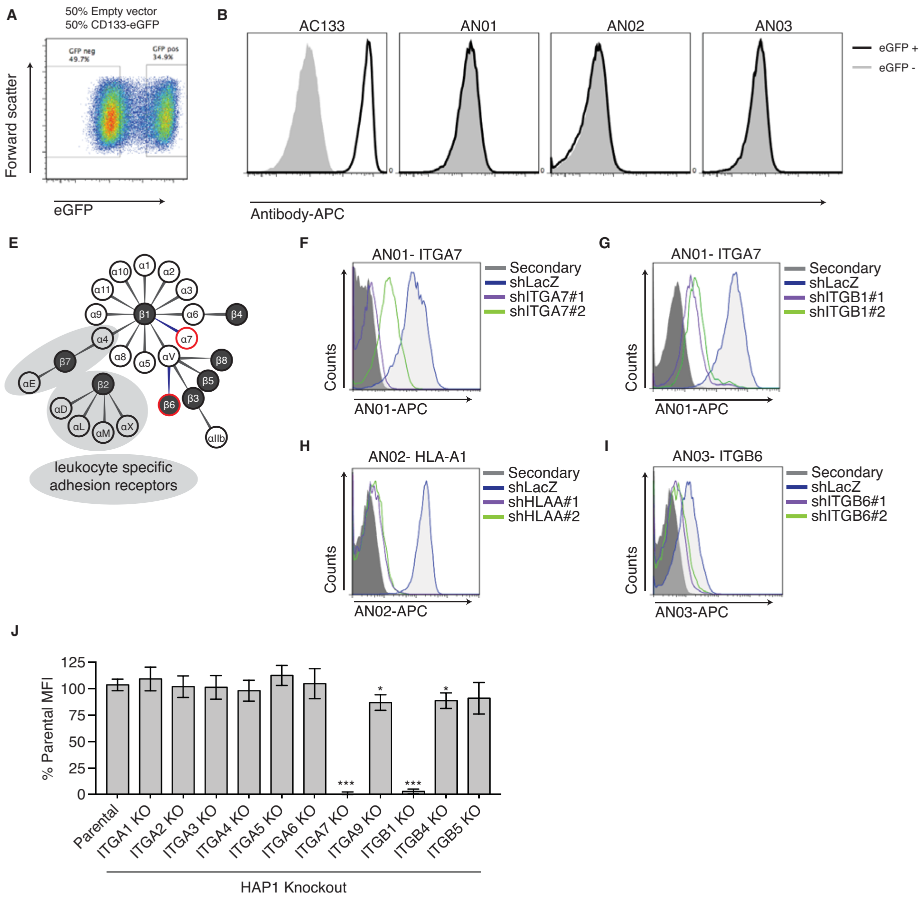
A
50% Empty vector
50% CD133-eGFP
GFP neg
49.7%
GFP pos
34.9%
Forward scatter
eGFP
B
AC133
AN01
AN02
AN03
eGFP +
eGFP -
Antibody-APC
E
α1
α2
α3
α6
β4
α10
α11
α9
β1
α4
α7
α8
α5
αV
β8
β7
β5
αE
β2
β6
β3
αD
αL
αM
αX
αIIb
leukocyte specific
adhesion receptors
F
AN01- ITGA7
Secondary
shLacZ
shITGA7#1
shITGA7#2
Counts
AN01-APC
G
AN01- ITGA7
Secondary
shLacZ
shITGB1#1
shITGB1#2
Counts
AN01-APC
H
AN02- HLA-A1
Secondary
shLacZ
shHLAA#1
shHLAA#2
Counts
AN02-APC
I
AN03- ITGB6
Secondary
shLacZ
shITGB6#1
shITGB6#2
Counts
AN03-APC
J
% Parental MFI
125
100
75
50
25
0
***
*
***
*
Parental
ITGA1 KO
ITGA2 KO
ITGA3 KO
ITGA4 KO
ITGA5 KO
ITGA6 KO
ITGA7 KO
ITGA9 KO
ITGB1 KO
ITGB4 KO
ITGB5 KO
HAP1 Knockout

Figure S2: *Identification of the antigens for AN01, AN02 and AN03.* **(A)** HEK293T cells stably expressing empty vector or CD133-eGFP fusion were mixed 50:50, stained with antibody and gated on eGFP expression. **(B)** Histograms show binding of each antibody in populations gated in A. **(C)** Histogram from a representative flow cytometry experiment assessing AN01, AN02 and AN03 binding to MAC-LS-2 breast cancer cell line. **(D)** Immunofluorescence staining of MAC-LS-2 live cell surface with AN01, AN02 or AN03. Blue is nucleus and Green is indicated antibody with anti-human488 secondary. **(E)** Schematic of integrin heterodimer pairing. **(F)** Histograms showing indicated antibody binding to POP92 cells treated with control shLacZ or two independent shRNA targeting *ITGA7*, **(G)** *ITGB1*, **(H)** *HLA-A*, and **(I)** *ITGB6*. **(J)** Bar chart illustrating the mean fluorescent intensity (MFI) staining of AN01 (ITGA7 antibody) on a panel of HAP1 cells with various integrin subunits knocked out (KO). Data is presented as % parental MFI from an in-tube parental control, error bars represent SD and significance was calculated with student's t-test. N=3 biological replicates.

Figure S3

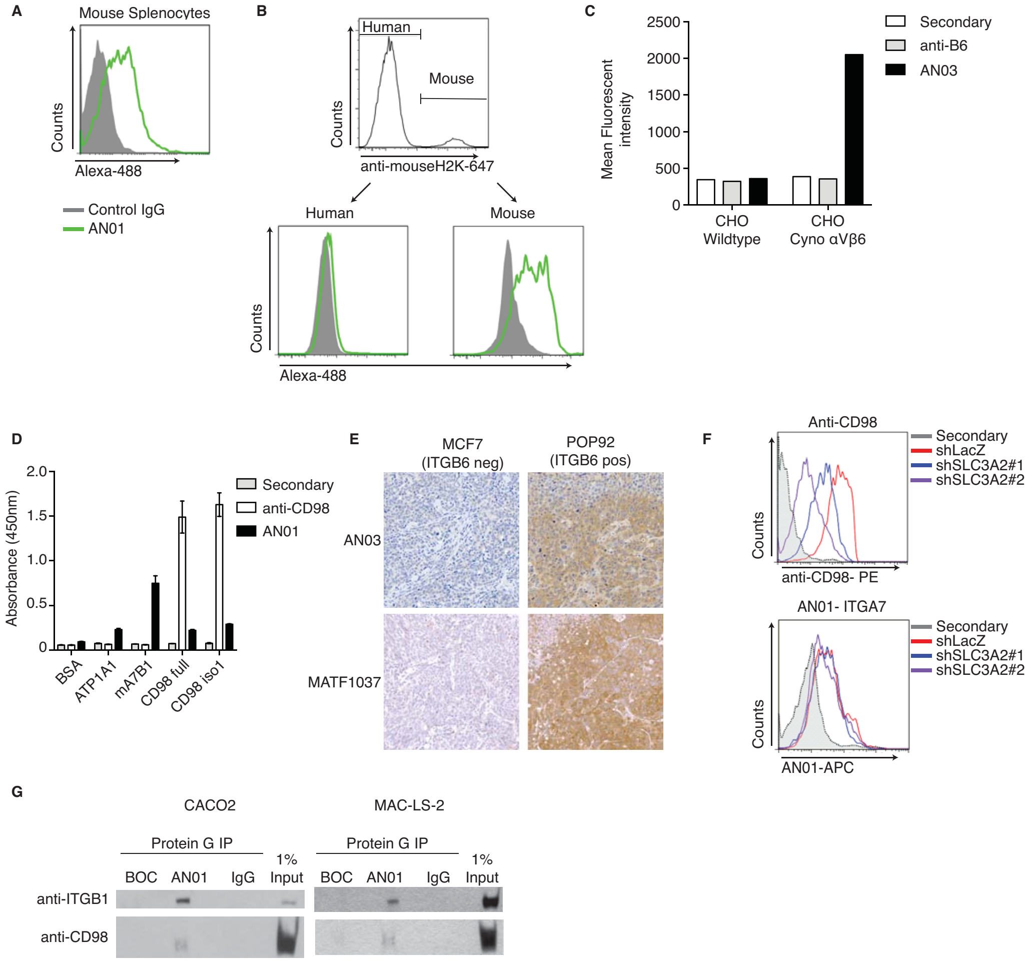
A
Mouse Splenocytes
Counts
Alexa-488
Control IgG
AN01
B
Human
Mouse
Counts
anti-mouseH2K-647
Human
Mouse
Counts
Alexa-488
C
Mean Fluorescent intensity
2500
2000
1500
1000
500
0
CHO Wildtype
CHO Cyno αVβ6
Secondary
anti-B6
AN03
D
Absorbance (450nm)
2.0
1.5
1.0
0.5
0
BSA
ATP1A1
mA7B1
CD98 full
CD98 iso1
Secondary
anti-CD98
AN01
E
MCF7 (ITGB6 neg)
POP92 (ITGB6 pos)
AN03
MATF1037
F
Anti-CD98
Secondary
shLacZ
shSLC3A2#1
shSLC3A2#2
Counts
anti-CD98- PE
AN01- ITGA7
Secondary
shLacZ
shSLC3A2#1
shSLC3A2#2
Counts
AN01-APC
G
CACO2
MAC-LS-2
Protein G IP
BOC AN01 IgG 1% Input
Protein G IP
BOC AN01 IgG 1% Input
anti-ITGB1
anti-CD98

Figure S3: *Antibody characterization.* **(A)** Histogram showing mouse splenocytes stained with AN01 or a control IgG. **(B)** POP92 isolated xenograft cells stained for with anti-mouseH2K and gated for human versus mouse cells. Histograms showing AN01 and control IgG binding to each population. **(C)** Parental CHO cell line and CHO cells overexpressing *M. fascicularis* integrin $\alpha V\beta 6$ stained with commercial $\beta 6$ antibody or AN03. **(D)** Bar chart from one ELISA experiment showing AN01 and commercial CD98 antibody binding to various recombinant proteins. Error bars are SD from 4 technical replicates. **(E)** IHC using AN03 ITGB6 antibody compared to commercial ITGB6 antibody MATF1037 on MCF7 breast tumour xenograft (ITGB6 negative) and POP92 xenograft (ITGB6 positive). **(F)** Histograms showing POP92 cells treated hairpins against *SLC3A2* (CD98 heavy chain) or shLacZ control stained with anti-CD98 (top) or AN01 (bottom). **(G)** Western blots showing immunoprecipitation (IP) of beads only control (BOC), AN01 IgG and control IgG from CACO-2 and MACL-S-2 cell lines probed for ITGB1 and CD98.
